# A meta-analytic review of multisensory imagery identifies the neural correlates of modality-specific and modality-general imagery

**DOI:** 10.3389/fnhum.2012.00285

**Published:** 2012-10-17

**Authors:** Chris McNorgan

**Affiliations:** Department of Communication Sciences and Disorders, Northwestern UniversityEvanston, IL, USA

**Keywords:** embodied cognition, imagination, imagery, modality-independent, modality-specific, semantic memory

## Abstract

The relationship between imagery and mental representations induced through perception has been the subject of philosophical discussion since antiquity and of vigorous scientific debate in the last century. The relatively recent advent of functional neuroimaging has allowed neuroscientists to look for brain-based evidence for or against the argument that perceptual processes underlie mental imagery. Recent investigations of imagery in many new domains and the parallel development of new meta-analytic techniques now afford us a clearer picture of the relationship between the neural processes underlying imagery and perception, and indeed between imagery and other cognitive processes. This meta-analysis surveyed 65 studies investigating modality-specific imagery in auditory, tactile, motor, gustatory, olfactory, and three visual sub-domains: form, color and motion. Activation likelihood estimate (ALE) analyses of activation foci reported within- and across sensorimotor modalities were conducted. The results indicate that modality-specific imagery activations generally overlap with—but are not confined to—corresponding somatosensory processing and motor execution areas, and suggest that there is a core network of brain regions recruited during imagery, regardless of task. These findings have important implications for investigations of imagery and theories of cognitive processes, such as perceptually-based representational systems.

Perception describes our immediate environment. Imagery, in contrast, affords us a description of past, future and hypothetical environments. Imagery and perception are thus two sides of the same coin: Perception relates to mental states induced by the transduction of energy external to the organism into neural representations, and imagery relates to internally-generated mental states driven by representations encoded in memory. Various forms of mental imagery have been implicated in a wide array of cognitive processes, from language comprehension (Bottini et al., 1994), to socially-motivated behaviors such as perspective taking (Ruby and Decety, 2001), to motor learning (Yágüez et al., 1998). Understanding the networks supporting imagery thus provides valuable insights into many behaviors.

## What are the neural substrates of modality-specific imagery?

Though representations generated through mental imagery clearly have perceptual analogs, a persistent question of the imagery literature concerns the extent to which imagery and perceptual processes overlap. Within the visual imagery domain, Kosslyn and Thompson (2003) analyzed contemporary neuroimaging studies to explain the lack of consistency with which studies demonstrate recruitment of early visual cortex during imagery. They showed that imagery was most likely to recruit early visual cortex when it requires attention to high-resolution detail, suggesting that perceptual processing during imagery depends on attention or processing level (Craik and Lockhart, 1972). The analogs question has been posed in the auditory and motor imagery domains, with some studies finding activation in primary sensorimotor areas (Wheeler et al., 2000; Hanakawa, 2002; Bunzeck et al., 2005) and others not (Zatorre and Halpern, 1996; Halpern and Zatorre, 1999; Vingerhoets et al., 2002).

In an early review of the imagery literature, Kosslyn et al. (2001) concluded from the auditory and motor imagery that dominated literature at the time, that “most of the neural processes that underlie like-modality perception are also used in imagery,” (p. 641). Subsequent study of imagery in other modalities and continuations of earlier lines of imagery study now afford a clearer picture of imagery across all sensory modalities and, importantly, of imagery in general. Moreover, recently developed analytic techniques now permit a more precise description of the perception-related processes underlying imagery. The present paper uses one such analytic technique to explore the body of modality-specific imagery literature with the overall aim of identifying the neural substrates of modality-specific and modality-general imagery. As will be discussed below, of particular importance is the question of whether modality-specific imagery recruits primary sensorimotor cortex as a rule. The resolution of this this question bears importantly on issues central to cognitive processes with which imagery is tightly bound.

## Imagery and perceptually-grounded representations: theoretical issues

Semantic memory—one's knowledge of the meaning of things—critically supports a wide array of cognitive processes, from language production and comprehension, to action planning. Of all cognitive processes, imagery and semantic processing are perhaps most closely related. Imagery regularly relies on previously organized and stored semantic information (Kosslyn et al., 1997) about the features to be imagined. A large body of literature makes the complimentary argument that the reactivation of perceptual representations—that is, imagery—underlies semantic retrieval. The assumption that imagery underlies semantic retrieval is the central premise of perceptually-based theories of cognition. The Perceptual Symbol System account (Barsalou, 1999) assumes that reactivation of perceptual representations (“perceptual simulations”) underlies semantic retrieval and provides one of the most recent and explicit accounts of the importance of imagery to semantic processing. Under this account, perceiving an object elicits a unique pattern of activation in primary sensorimotor cortices encoding salient perceptual properties of that object. Perceptually-based theories argue that encoding and retrieving these activations within the perceptual system naturally permits high-fidelity perceptually-rich representations. Similar ideas underlie Warrington and McCarthy's sensory/functional theory (Warrington and McCarthy, 1987), and Paivio's dual coding theory (Paivio, 1971), which explicitly argues that abstract propositional and (visual) imagery representations comprise concept knowledge.

Full elucidation of the assumptions and criticisms of a perceptually-grounded system are beyond the scope of this article, but have been given extensive consideration elsewhere (Barsalou, 1999; Simmons and Barsalou, 2003). One advantage of perceptually-grounded models is that they arguably overcome the reverse inference problem (Poldrack, 2006), which is the neuroimaging equivalent of the symbol grounding problem (Harnad, 1990). The symbol grounding problem describes the circularity inherent in relating arbitrary symbols to an equally arbitrary symbol system. The solution Harnad proposes is for one symbolic system to be non-arbitrary—that is, to be grounded in an external physical system. Because primary sensory cortices contain populations driven by external physical systems, the perceptual system provides the grounding required to understand those cognitive systems that interact with it. For example, patterns of activations within olfactory cortex reflect detection of particular smells. Olfactory imagery and knowledge retrieval might engage a wide network of brain areas related to any number of cognitive processes. However, if imagery is perceptually-grounded, one would additionally expect an involvement of the corresponding sensory cortex. Whatever other brain regions may contribute toward olfactory imagery, it is relatively straightforward to argue activity within olfactory cortex is part of an olfactory representation.

## Imagery and perceptually-grounded representations: methodological issues

The strong theoretical ties between perceptually-based semantic theories and imagery suggest that a thorough understanding of the former requires an understanding of imagery. It is important to reiterate that perceptually-based representational theories assume that semantic representations are rooted in imagery, rather than perception *per se*. Nonetheless, a common practice is to localize these perceptually-based representational systems using perceptual tasks. For example, Simmons et al. (2007) investigated color knowledge retrieval within color-sensitive visual cortex localized using a modified Farnsworth-Munsell 100-Hue Task (Farnsworth, 1957).

The demonstration of a common neural basis underlying perception and modality-specific semantic knowledge provides compelling support for perceptually-based theories. Such findings support a strong version of a perceptually-grounded semantic system—that is, that perceptual processing is implied by semantic retrieval. The approach of using primary sensory cortex to define these representational areas has some limitations, however. First, the choice of localizer task is not a trivial consideration, and may impact the ability to detect the true extent of the modality-specific region. For example, recruitment of color-selective areas has been shown to be modulated by attention (Beauchamp et al., 1999), and thus different perceptual localizer tasks may give different estimates of the corresponding perceptual areas. Second, multiple localization tasks and specialized delivery apparatus required for some perceptual tasks may be impractical for investigations of multiple representational modalities. Even when primary sensory regions are well-defined, there remains one important consideration: Semantic encoding and retrieval processes are assumed to be rooted in imagery. Thus, to the extent that the network supporting imagery extends beyond primary somatosensory perceptual areas, important imagery-related contributions to semantic encoding and retrieval may be overlooked. Thus, an understanding of the neural substrates underlying imagery provides critical insight into the organization of the semantic system, and can guide investigations of representational systems.

## The ALE meta-analytic technique

An empirically-driven characterization of the neural correlates of modality-specific and modality-general imagery processes has been made possible in recent years by the development of meta-analytic techniques for assessing neuroimaging data. Techniques such as Activation Likelihood Estimation (ALE) (Chein et al., 2002; Turkeltaub et al., 2002) and Multilevel Kernel Density Analysis (MKDA) (Wager et al., 2007) allow the application of statistical measures to the literature to assess the reliability with which an effect is demonstrated in a particular brain area. In short, these methods permit an empirical test of consensus within a body of neuroimaging literature. A detailed explanation of the advantages and underlying statistics behind voxel-based meta-analytic approaches was presented by Laird et al. (2005). Briefly, these approaches examine the activation foci reported for a common contrast among multiple studies. Statistical tests on these data (e.g., chi-square analyses, Monte Carlo simulations) provide quantifiable, statistically-thresholded measures of the reliability of activation for a given contrast within a given region. As with other meta-analytic techniques, these approaches importantly highlight commonalities among studies, and minimize idiosyncratic effects. The ALE approach has been used in recent years to examine representational knowledge in the semantic system in general (Binder et al., 2009) and for more specific representational knowledge about categories such as tools and animals (Chouinard and Goodale, 2010). The utility of this approach in identifying important networks within these domains suggests it may be similarly useful in the conceptually-related imagery domain.

What follows is an ALE analysis of the neuroimaging literatures in modality-specific imagery across visual, auditory, motor, tactile, olfactory, and gustatory modalities. These analyses provide a descriptive survey of the imagery literature and were intended to meet three main goals: First, to identify the brain areas recruited during imagery, regardless of modality. Second, to identify within each modality the brain regions associated with modality-specific imagery with particular attention to the extent to which primary sensorimotor perceptual regions are recruited. Finally, various sub-processes are carried out by different and well-defined populations of neurons tuned for processing color, form and motion during visual perception. The number of studies investigating corresponding subtypes of visual imagery provides an opportunity to investigate whether evidence for a similar organization can be found during visual imagery.

## Materials and methods

Searches for candidate imagery studies were conducted in the PubMed and Google Scholar databases for fMRI and PET studies related to imagery or sensory-specific imagery (e.g., “gustatory imagery,” “taste imagery”). Iterative searches within the citations among candidate imagery studies located additional candidate imagery studies with the intention of creating a comprehensive list of studies explicitly examining imagery or imagery-like tasks. For purposes of this study, imagery-like tasks were defined as those for which the retrieval of perceptual information from long term memory was required. These tasks were framed as perceptual knowledge retrieval by study authors and extensively cited or were cited by explicit studies of imagery. As discussed above, perception-based theories of knowledge representations are explicitly rooted in imagery (Paivio, 1971; Warrington and McCarthy, 1987; Barsalou, 1999), and a large body of literature supports the hypothesis that imagery underlies perceptual knowledge retrieval. Consequently, in many cases, similar tasks were used by different authors to investigate perceptual knowledge retrieval and imagery [e.g., color feature verification used by Kellenbach et al. (2001) and color feature comparison used by Howard et al. (1998)]. ALE measures concordance among reported activations; therefore heterogeneity among studies should lead to a reduction in power, rather than inflation of type I error. To the extent that perceptual knowledge retrieval does not involve imagery processes, inclusion of perceptually-based knowledge studies should therefore lead to slightly more conservative estimates of imagery activation. These studies comprised a small minority of the overall body of literature surveyed, however, so any such conservative bias should be rather small. For these reasons, these inclusionary criteria were deemed appropriate. Studies investigating special populations (e.g., synaesthetes, neurological patients) were excluded, as were those that did not conduct whole brain analyses or report coordinates in stereotactic space for significant modality vs. baseline contrasts. The studies included in the present analysis are listed in Table 1.

**Table 1:** **Studies used in the imagery meta-analysis**.

**Modality**	**First author**	**Year**	**Imagery task**	**Baseline**
AUD^*^	Belardinelli	2009	Imagine performing sensory action	Listen to sentence designating abstract concept
AUD	Bunzeck	2005	Imagined sounds corresponding to movie	Passive viewing of scrambled scene
AUD	Halpern	1999	Imagine continuation of tone sequence	Passive listening to tones
AUD^*^	Kellenbach	2001	Retrieval of sensory specific object knowledge concerning color, sound, size	Visual search for X in unrelated letter string
AUD^*^	Kiefer	2008	Lexical decision on stimuli with and without auditory features	Implicit/rest
AUD	Nyberg	2000	Recall sounds paired with textual cue	Implicit/rest
AUD	Wheeler	2000	Recall studied complex picture or sound	Opposite modality recall
AUD	Yoo	2001	Imagine recorded chord in response to cue	Implicit/rest
GUS^*^	Belardinelli	2009	Imagine performing sensory action	Listen to sentence designating abstract concept
GUS	Kikuchi	2005	Imagine taste of strong-tasting pictured foods	Viewing colored balls
GUS	Kobayashi	2004	Taste recall for pictured food items	Implicit/rest
GUS	Small	2003	Same/different judgments of pictured foods vs. locations	Passive viewing of locations
GUS	Veldhuizen	2007	Detection of taste in a tasteless solution	Passive swallowing
MTR^*^	Belardinelli	2009	Imagine performing sensory action	Listen to sentence designating abstract concept
MTR	Canessa	2007	Judgments whether items are manipulated using the same action	Implicit/rest
MTR	Creem-Regehr	2007	Mental rotation of self(motor) or other (visual)	Implicit/rest + no rotation
MTR	Dechent	2004	Imagined execution of trained finger tapping sequence	Visual imagery of scene
MTR	Guillot	2009	Imagined motor execution	Passive tone listening
MTR	Hanakawa	2002	Imagined execution of trained finger tapping sequence	Fixation
MTR^*^	Hauk	2004	Reading action words associated with specific body parts (e.g., “KICK”)	Fixation
MTR	Johnson	2002	Imagined grip	Foil trials
MTR	Nyberg	2001	Imagined execution of actions	Implicit/rest
MTR	Servos	2002	Imagined execution of motor sequence	Visual object imagery
MTR^*^	Simmons	2003	Property verification	Lexical decision
MTR	Vingerhoets	2002	Mental rotation of tools and hands to make same/different judgments	Passive viewing (non-rotated pictures)
OLF^*^	Belardinelli	2009	Imagine performing sensory action	Listen to sentence designating abstract concept
OLF	Djordjevic	2005	Imagined odors	Odor detection in the absence of odor
OLF	Gottfried	2004	Recall of odor paired with object pictures during training	View picture without associated odor
OLF	Plailly	2012	Odor imagery	Implicit/rest
OLF	Yeshurun	2009	Recall remembered smell	Implicit/rest
TAC^*^	Belardinelli	2009	Imagine performing sensory action	Listen to sentence designating abstract concept
TAC^*^	Newman	2005	Haptic/form judgments on pairs of concrete object names	Implicit/rest
TAC	Yoo	2003	Imagined tactile stimulation	Implicit/rest
VCO	Hsu	2011	Color word similarity judgment	Implicit/rest
VCO	Hsu	2012	Relative luminance decision on chromatic/achromatic object names	Evaluative judgments on abstract concepts
VCO^*^	Kellenbach	2001	Retrieval of sensory specific object knowledge	Visual search for X in unrelated letter string
VCO	Sack	2002	Mental clock task; color and angle judgments	Implicit/rest
VFO^*^	D'Esposito	1997	Visualize named concrete objects	Abstract concept
VFO	Ganis	2004	Visualize a line drawing	Implicit/rest
VFO	Gulyás	2001	Visualize capital letters from a known passage of text	Implicit/rest
VFO	Ishai	2000	Visualize recently studied or famous faces	Passively view letter strings
VFO^*^	Kellenbach	2001	Retrieval of sensory specific object knowledge	Visual search for X in letter string
VFO	Kosslyn	1993	Visualize uppercase block letters	Response to unrelated target
VFO	Kosslyn	1995	Visual form judgments on imagined line drawings	Passive listening
VFO	Kosslyn	1997	Visualize uppercase block letters	Response to target grid element
VFO^*^	Mellet	1996	Three-dimensional object visualization	Passive listening/rest
VFO^*^	Newman	2005	Haptic/form judgments on pairs of concrete object names	Implicit/rest
VFO^*^	Oliver	2009	Property verification	Lexical decision
VFO	Sack	2002	Mental clock task; color and angle judgments	Implicit/rest
VFO	Thompson	2001	Compare visualized and displayed patterns	Response to unrelated auditory cue
VFO	Trojano	2000	Comparing visualized clock faces	Numerical judgment
VFO	Yomogida	2004	Object imagery and synthesis	Implicit/rest
VMO	Alivisatos	1997	Mental rotation	Discrimination of retters/rumbers
VMO	Barnes	2000	Mental rotation and linear translation	Implicit/rest
VMO	Creem-Regehr	2007	Mental rotation of self (MTR) or other (VMO)	Implicit/rest + no rotation
VMO	de Lange	2005	Judge laterality of left/right hands	Visual imagery
VMO	Goebel	1998	Imagine previously studied moving stimuli	Implicit/rest
VMO	Guillot	2009	Visualized motor execution from 1st person perspective	Passive tone listening
VMO	Jordan	2001	Mental rotation	Same/different/ numerosity judgments on static figures
VMO	Kaas	2010	Imagine moving ball	Passively listening to unrelated tone
VMO	Slotnick	2005	Mental rotation	Passively attend to display

Note: “

* ” denotes experiment with a semantic component; AUD, auditory, GUS, gustatory; MTR, motor; OLF, olfactory; TAC, tactile; VCO, visual-color; VFO, visual-form; VMO, visual-motion.

Imagery vs. low-level baseline contrasts were categorized with respect to one of eight modality conditions: Auditory, Tactile, Motor, Olfactory, Gustatory, Visual-Form, Visual-Color, and Visual-Motion. Modality categorizations were generally straightforward to determine (e.g., taste recall clearly relating to gustatory imagery), though classification of visual imagery subtypes required careful consideration of the task, stimuli and baseline contrasts used. One study (Roland and Gulyás, 1995) required participants to recall both the colors and geometric description of colored geometric patterns. The remaining visual form studies used monochromatic stimuli. The relative scarcity of color imagery studies, and the saliency of both form and color information in the task motivated the inclusion of this study in both modalities. Despite this single commonality, the ALE maps for these two modalities did not resemble one another.

The focus on the lowest-level baseline contrast was mandated by the fact that it alone was included across all imagery studies. Though the baseline task varied among studies, ranging from rest baselines to passive viewing baselines controlling for other modalities (e.g., passive viewing of scrambled scenes for auditory imagery) or within-modality (e.g., passive viewing of letter strings for form imagery), no particular baseline task dominated any modality. Implicit or resting-baselines were used in approximately 40% of studies, nearly all of which employed tasks requiring no overt response on the part of the participant. The remaining studies employed somewhat more complex baseline tasks generally designed to account for attention or response processes (e.g., those associated with button presses) under the assumption of cognitive subtraction. Direct contrasts between perception- and imagery-related activity tend to show reduced activity in primary perceptual areas for imagery relative to perception (e.g., Ganis, 2004). Care was thus taken to ensure that baselines involving a sensorimotor component excluded activity only in modalities of non-interest. For example, the detection of taste within a tasteless solution (Veldhuizen et al., 2007) likely involves motor activity in the planning and execution of passing the solution over the tongue and swallowing. The baseline task used in that study involved swallowing the solution without making a taste judgment. Under the assumption of pure insertion, the contrast between the two tasks should reveal activations associated only with the gustatory judgment (but see Friston et al., 1996 for a critique of the logic of cognitive subtraction). In the analyses that follow, however, the lack of systematicity among active baseline tasks somewhat mitigates concerns about the validity of cognitive subtraction. Aggregated across studies, imagery-related activations should be more reliable than those related to particular baseline choices, just as random-effects analyses across participants distinguish the influence of an experimental manipulation from noise. Though the complexity of baseline task was generally commensurate with that of the experimental task across studies, baseline complexity was investigated in detail in the general imagery analysis, where the numbers of studies permitted such an analysis. The reduced number of studies available for individual modalities, however, precluded such an analysis within each modality.

Concordance among imagery vs. baseline activation foci reported across the neuroimaging literature was analyzed using a widely used activation likelihood estimate (ALE) meta-analytic approach (Eickhoff et al., 2012). Analyses were performed using GingerALE 2.1 (http://brainmap.org/ale/). Correction for multiple comparisons was performed using a false-discovery rate (FDR) threshold of *pN* < 0.05. GingerALE reports the number of voxels meeting the selected FDR threshold within each ALE map. Except where noted, a cluster size threshold, equal to the FDR rate times the number of suprathreshold voxels, was applied to each map (hereafter *extent-thresholded clusters*). For example, if 1000 voxels reached a FDR threshold of 0.05, then the expected number of false positives within that ALE map would be 50. A cluster size threshold of 50 in this example ensures that no extent-thresholded cluster would consist entirely of false positives. Because the number of FDR-significant voxels varied by modality, this approach resulted in different cluster thresholds across modalities. It should be noted, however, that imagery-related clusters were analyzed independently and with respect to sensorimotor ROIs rather than with each other (see below). Thus, these differences had little to bear on the results that follow, other than to increase the confidence with which conclusions can be drawn about the meaningfulness of any given extent-thresholded cluster for that analysis.

### ROI definition and overlap analysis

The question of whether modality-specific imagery activates primary sensorimotor cortex was addressed within each modality by assessing the overlap between extent-thresholded ALE clusters and the primary sensorimotor ROI defined for each modality. ROIs were drawn from several publicly available anatomical atlases. The source(s) for each ROI are indicated in each modality analysis. Multiple atlases were necessitated by the fact that no single atlas contained ROI definitions corresponding to all modalities included in the present analysis. In some cases, different atlases contained different definitions of the same region. When a given anatomical region was defined in exactly one atlas, that definition was taken as the ROI; when multiple atlases defined the same region, the intersection (i.e., only those voxels common to all definitions) was taken as the ROI. This atlas-based approach was intended to arrive at a set of ROIs that are easily reproducible and for which there should be general agreement are representative of the corresponding sensorimotor cortices.

The degree of overlap was assessed for each ROI by determining whether the number of voxels in the extent-thresholded ALE clusters overlapping with a given ROI reached an overlap criterion. The overlap criterion was set independently for each ROI using 3dClustSim (available as part of the AFNI fMRI analysis package, available at http://afni.nimh.nih.gov/afni/download). Briefly, 3dClustSim calculates cluster size threshold (k) for false positive (noise-only) clusters at specified uncorrected alpha level. Though the ALE analyses used FDR corrected alpha thresholds, the equivalent voxel-wise alpha threshold for each ALE map is available in the GingerALE output. 3dClustSim carries out a user-specified number of Monte Carlo simulations of random noise activations at a particular voxel-wise alpha level within a masked brain volume. Ten thousand such simulations for each ALE map were used for this study. The number of simulations in which clusters of various sizes appear within the volumetric mask is tallied among these simulations. These data are then used to calculate size thresholds across a range of probability values for that region. For example, in a specified volume using a voxel-wise alpha of 0.001, if clusters of size 32 mm^3^ or greater appear in 50 of 10,000 iterations by chance, this correspond to a *p* < 0.05 cluster-level significance threshold. In other words, within the specified volume using a voxel-wise alpha of 0.001, clusters exceeding 32 mm^3^ are unlikely to occur by chance. To be clear, the cluster thresholds calculated using 3dClustSim was used to calculate an overlap criterion for each ROI, and not as an additional ALE cluster thresholding step. To the author's knowledge, no previous meta-analysis of neuroimaging data has attempted to qualify overlap between ALE clusters and a priori ROIs. However, the cluster size threshold approach is widely used to test statistical significance of clusters in conventional ROI analyses. That is, size thresholding is often used to determine whether a cluster of a particular size occurring within a given ROI is statistically significant. The present analysis had identical requirements, thus it was deemed to be an appropriate metric of overlap significance. A benefit of this approach when considering different ROIs is that it naturally takes into account differences in ROI extents: Larger sensorimotor ROIs require correspondingly greater overlap with imagery clusters for the overlaps to reach statistical significance.

Finally, it is important to note that the following analyses identify concordance of activation across studies within each modality, rather than contrast modalities directly. That is, they do not identify regions of activation unique to imagery in a particular modality. There are regions for which only studies of imagery for one modality converges (e.g., gustatory cortex activation apparent only for gustatory imagery studies). Nonetheless, the following results do not speak to whether one imagery modality recruits a particular region more than any other imagery. Inter-modal contrasts were not performed for two reasons: First, such contrasts address the question, not of what regions are implicated in a particular type of imagery, but what regions are implicated more for that type of imagery than any other. Networks defined by such contrasts would thus be more exclusive, and reducing the usefulness of these analyses to those interested in a non-comparative description of imagery for a particular modality. Second, there is a practical problem imposed by the disparity between the frequencies with which imagery in each sensorimotor modality has been investigated. This disparity would plausibly skew any such comparisons and generate networks driven by a single modality. When analyses are restricted to within-modality, however, differences with respect to numbers of studies are not problematic: a coherent network can be identified from relatively few studies, provided they are mutually consistent. Though modality-specific activations are not explicitly contrasted, crossmodal overlap between clusters is noted where it occurs.

## Results

The results are presented in order of generality. The first analysis identifies those regions consistently active relative to baseline in neuroimaging studies of imagery across all modalities. The eight subsequent analyses identify regions consistently active relative to baseline in modality-specific imagery for each of 5 sensorimotor modalities and 3 subtypes of visual imagery. All coordinates are reported in MNI standard space.

### General imagery network

**Figure 1:**
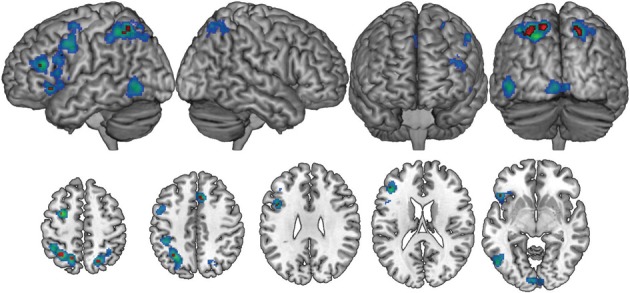
**The general imagery network (cool colors) was primarily left-lateralized, with bilateral activations in superior parietal regions.** A conjunction analysis of studies employing complex and resting state baselines found nine clusters (red) within the general imagery network that were active across all imagery conditions, regardless of baseline task.

A statistical threshold of *pN* < 0.01 (FDR corrected) and a minimum cluster size threshold of 800 mm^3^ was used for the general imagery analysis. One thousand hundred and three foci from 84 contrasts involving 915 participants contributed toward these results. Nine primarily left-lateralized clusters reached the significance threshold (Table 2, Figure 1). These activations were found in bilateral dorsal parietal, left inferior frontal and anterior insula regions.

**Table 2:** **Weighted centers of clusters in the general imagery ALE analysis**.

	**Region**	**BA**	***x***	***y***	***z***	**Volume**
All studies	L Superior/Inferior Parietal Lobule/Precuneus	7/40	−28	−56	51	10544
	L Inferior/Middle Frontal Gyrus/Precentral Gyrus	9/47/6	−43	14	18	7216
	R Precuneus/Superior Parietal Lobule	7	22	−63	54	3216
	L Middle Occipital Gyrus	37	−51	−63	−5	2320
	L Middle Frontal Gyrus	46	−41	33	19	2064
	L Middle Frontal Gyrus	6	−28	−1	55	1904
	L Putamen/Caudate/Insula		−25	0	4	1448
	R Medial Frontal Gyrus	6	6	21	45	1376
Conjunction of contrasts vs. rest and non-rest baselines	L Superior/Inferior Parietal Lobule	7/40	−30	−56	52	620
	L Superior Parietal/Precuneus	5/7	−16	−62	54	320
	R Superior Occipital/Parietal Gyrus	7	20	−66	54	150
	R SMA/Med Superior Frontal Gyrus/Cingulum	32/8	6	20	44	90
	L Precentral/Middle Frontal Gyrus	6	−30	0	56	60
	L Inferior Parietal Lobule	40	−38	−38	46	20
	L Precentral/Inferior Frontal Gyrus	44/48	−42	10	28	20
	L Inferior Frontal Gyrus	38/47	−48	24	−6	20
	L Inferior Frontal Gyrus	45	−44	34	18	10

L, Left; R, Right; SMA, Supplementary Motor Area; Med, Medial; BA, Brodmann area. Volume is measured in mm^3^. Coordinates reflect standard MNI space.

As indicated earlier, one advantage of meta-analytic techniques is that random-effects analyses minimize spurious effects attributable to idiosyncratic experimental design decisions among studies (e.g., choice of baseline) and highlight commonalities among them (e.g., choice of imagery modality). Imagery vs. baseline contrasts in the ALE analyses involved two broad classes of low-level baseline tasks: The resting state baseline tasks are assumed to be homogeneous across the 33 contrasts that used them. The non-resting state baseline tasks used across the remaining 50 contrasts were more varied, typically involving passive perceptual control conditions (for non-target modalities) or foil trials. Because ALE is sensitive to activation consistencies, it was plausible that baseline-related (rather than strictly imagery-related) networks may emerge in the ALE statistics. This concern was conservatively addressed by a conjunction analysis of resting-baseline vs. non-resting-baseline studies. The significance threshold was maintained at *pN* < 0.01 (FDR corrected) for both baseline conditions, though no cluster extent threshold was used (the resulting false discovery rate was 0.0001). The conjunction analysis found nine clusters, primarily in bilateral dorsal parietal and left inferior frontal regions that were active across all imagery modalities for all baseline conditions (Table 2). These results are suggestive of a core imagery network, though the extent of activation beyond this core network presumably depends baseline task. As the remaining analyses indicate, these activations also depend on imagery modality.

### Auditory imagery

**Figure 2:**
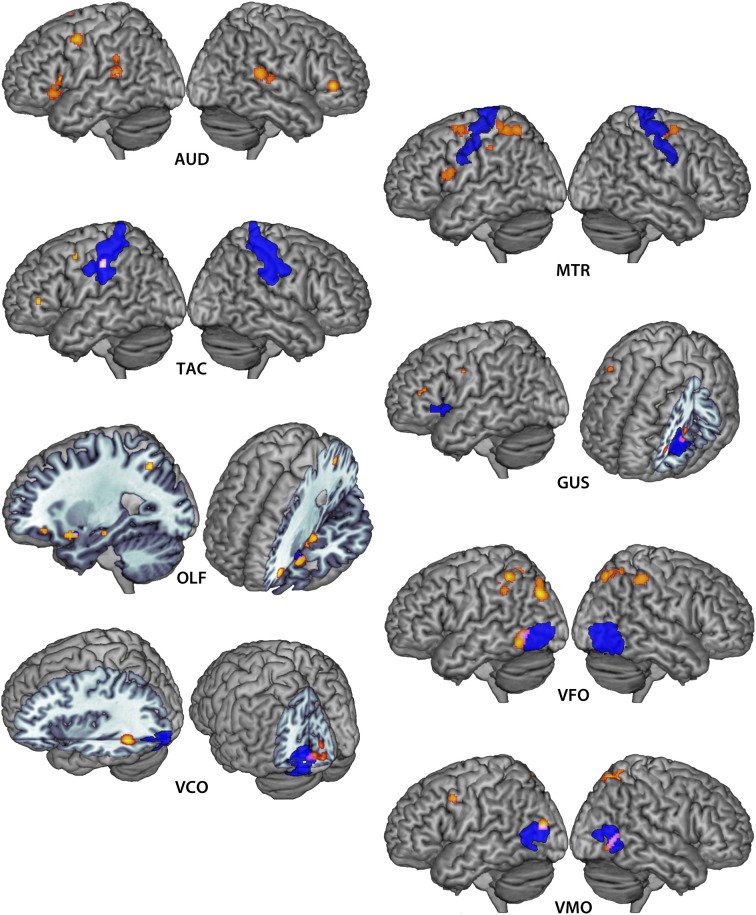
**Extent-thresholded clusters of voxels reaching *pN* < 0.05 significance in the auditory (AUD), motor (MTR), tactile (TAC), gustatory (GUS), olfactory (OLF), visual form (VFO), visual color (VCO) and visual motion (VMO) imagery ALE maps are depicted in warm values.** Corresponding primary sensorimotor cortices are depicted in violet. Overlapping regions are depicted in pink/white.

Minimum cluster size threshold in the ALE analysis of auditory imagery studies was set at 632 mm^3^. Ninety-three foci from 11 experiments involving 127 participants contributed toward these results. For the purposes of this analysis, primary auditory cortex was defined by the AAL template definition of Heschl's Gyrus within the MRIcroN software package (http://www.mccauslandcenter.sc.edu/mricro/mricron/index.html). Ten clusters were reliably associated with auditory imagery at a statistical threshold of *pN* < 0.05 (FDR corrected) (Table 3). No cluster overlapped with primary auditory cortex. Seven of eleven auditory imagery experiments reported activation peaks within two ALE clusters bilaterally overlapping secondary auditory cortex (planum temporale), indicating reliable activation of these areas during auditory imagery (Figure 2). Bilateral activations of inferior frontal cortex were also apparent. Because the imagery tasks used across auditory imagery experiments were non-linguistic in nature (e.g., tone imagery), involvement of Broca's area in auditory imagery was not readily attributable to language-related phonological processing.

**Table 3:** **Weighted centers of significant clusters in the auditory, motor, tactile, and gustatory imagery ALE analyses**.

**Modality**	**Region**	**BA**	***x***	***y***	***z***	**Volume**
Auditory	R Superior Temporal Gyrus	22	64	−30	9	2056
	L Inferior Frontal Gyrus	47	−48	24	−5	1360
	L Putamen/Globus Pallidus		−21	−1	4	1136
	L Inferior Frontal Gyrus	44	−51	17	9	1104
	L Superior Temporal Gyrus	22	−60	−38	15	1088
	L Precentral Gyrus	4	−52	1	47	920
	L Inferior Parietal Lobule	40	−58	−38	28	664
	R Inferior Frontal Gyrus	46	56	38	2	648
	L Medial Frontal Gyrus	6	−1	−14	53	640
	L Superior Frontal Gyrus	6	−8	1	69	640
Motor	L Inferior/Superior Parietal Lobule	40/7	−37	−43	53	4464
	L Precentral/Superior Frontal Gyrus	6	−26	−1	56	3584
	R Middle Frontal/Precentral Gyrus	6/4	33	−3	56	1000
	L Inferior Frontal Gyrus	44/45	−57	10	17	976
	L Medial Frontal Gyrus	6	2	5	56	768
Tactile	L Postcentral Gyrus	2	−56	−24	43	344
	L Inferior Frontal Gyrus	46	−51	39	6	96
	L Precentral Gyrus	6	−52	3	50	88
Gustatory	L Middle Frontal Gyrus	46	−41	35	16	272
	L Claustrum		−30	0	12	96
	L Claustrum		−39	0	6	80
	L Precentral Gyrus	6	50	−6	32	80
	R Red Nucleus		3	−27	−15	64
	L Insula		−39	9	3	64
	L Middle Frontal Gyrus	10	−33	43	3	64
	L Precentral Gyrus	6	−50	−4	34	64
	R Middle Frontal Gyrus	6	45	3	51	64
	L Superior Frontal Gyrus	6	−3	15	51	64

L, Left; R, Right; BA, Brodmann area. Volume is measured in mm^3^. Coordinates reflect standard MNI space.

### Motor imagery

Minimum cluster size threshold in the ALE analysis of motor imagery studies was set at 712 mm^3^. One hundred and fifty seven foci from 13 experiments involving 137 participants contributed toward these results. For the purposes of this analysis, motor cortex was defined by the Brodmann area 4 definition within the MRIcroN software package. Five clusters were reliably associated with motor imagery at a statistical threshold of *pN* < 0.05 (FDR corrected) (Table 3; Figure 2). Recruitment of primary motor cortex in motor imagery was not apparent in either hemisphere, though three clusters overlapped to a large extent (right: 222 mm^3^; left superior: 608 mm^3^; left inferior: 72 mm^3^) with premotor cortex. The posterior-most motor imagery cluster, centered at (*x* = −37, *y* = −43, *z* = 53), did overlap substantially with the tactile imagery ROI. The overlapping region was centered at (*x* = −38, *y* = −37, *z* = 53: 1351 mm^3^). The 3dClustSim simulations determined that the volume of this overlapping region corresponded to a cluster size corrected threshold of *p* < 0.001 within the primary somatosensory cortex ROI.

### Tactile imagery

Minimum cluster size threshold in the ALE analysis of tactile imagery studies was set at 88 mm^3^. Forty-nine foci from four experiments involving 44 participants contributed toward these results. For the purposes of this analysis, primary somatosensory cortex was defined by the union of the Brodmann area 1, 2, and 3 definitions within the MRIcroN software package. Three left-lateralized clusters were reliably associated with motor imagery at a statistical threshold of *pN* < 0.05 (FDR corrected) (Table 3; Figure 2). Recruitment of primary sensorimotor cortex was apparent in the cluster centered at (*x* = −56, *y* = −24, *z* = 43: 344 mm^3^), which overlapped entirely with primary somatosensory cortex. The 3dClustSim simulations determined that the volume of this overlapping region corresponded to a cluster size corrected threshold of *p* < 0.001 within the primary somatosensory cortex ROI. No tactile imagery ALE cluster overlapped with the primary motor cortex ROI.

### Gustatory imagery

Minimum cluster size threshold in the ALE analysis of gustatory imagery studies was set at 45 mm^3^. Note that this cluster size threshold was smaller than the GingerALE-recommended minimum threshold for this dataset. The 3dClustSim analysis determined that 45 mm^3^ clusters of size would occur by chance within the gustatory ROI with a probability of 0.05. This reduced cluster threshold permitted the detection of clusters that would reach corrected-level significance in the ROI. Fifty-three foci from five experiments involving 63 participants contributed toward these results. For the purposes of this analysis, gustatory cortex was defined by the AAL template definition of bilateral frontal operculum and anterior bilateral insula (*y* > 7, corresponding to the anterior third of the volume of the AAL template insula definition). The ALE analysis of all gustatory imagery studies found nine clusters that were reliably associated with gustatory imagery (Table 3; Figure 2). There was evidence for left-lateralized recruitment of gustatory cortex in gustatory imagery: One cluster, centered at (left: *x* = −39, *y* = 9, *z* = 3: 64 mm^3^), was overlapped completely by the gustatory cortex definition. The 3dClustSim simulations determined that the volume of this overlapping region corresponded to a cluster size corrected threshold of *p* < 0.05 within the primary gustatory cortex ROI.

### Olfactory imagery

Minimum cluster size threshold in the ALE analysis of olfactory imagery studies was set at 136 mm^3^. Fifty-one foci from five experiments involving 80 participants contributed toward these results. Olfactory cortex was defined by the AAL template definition of bilateral piriform cortex. The ALE analysis of all olfactory imagery studies found four clusters that were reliably associated with olfactory imagery (Table 4; Figure 2). There was overlap, centered at (*x* = −25, *y* = 8, *z* = −16: 14 mm^3^), between olfactory cortex and the third largest cluster centered at (*x* = −28, *y* = 11, *z* = −17). The 3dClustSim simulations determined that the volume of this overlapping region corresponded to a cluster size corrected threshold of *p* < 0.001 within the primary olfactory cortex ROI.

**Table 4:** **Weighted centers of significant clusters in the olfactory, visual form, visual color, and visual motion imagery ALE analyses**.

**Modality**	**Region**	**BA**	***x***	***y***	***z***	**Volume**
Olfactory	L Anterior Cingulate	32	−22	38	−11	376
	L Hippocampus	28	−23	−18	−17	280
	L Insula/Amygdala	34/38	−28	11	−17	256
	L Superior Parietal Lobule	7	−24	−61	46	256
Vis Form	R Precuneus	7	22	−67	51	3960
	L Inferior/Superior Parietal Lobule/Supramarginal/Angular Gyrus	40	−38	−50	48	3352
	R Lingual Gyrus	17/18	3	−93	−5	2104
	L Superior Occipital Gyrus/Precuneus	39	−33	−78	38	1760
	R Medial Frontal Gyrus	6/32	2	18	47	1424
	R Inferior Parietal Lobule/Supramarginal Gyrus	40	45	−38	46	1400
	L Inferior Temporal Gyrus	20/37	−55	−59	−8	1344
Vis Color	L Lingual Gyrus	18	−13	−85	−4	976
	L Fusiform Gyrus	37	−39	−51	−12	832
Vis Motion	R Precuneus	7	19	−60	61	2120
	R Middle Temporal Gyrus	37	47	−63	0	704
	L Middle Occipital Gyrus	19	−43	−81	16	528
	L Precuneus	31/7	−18	−76	36	528
	L Precuneus	7	−16	−58	59	456
	L Middle Frontal Gyrus	6	−46	7	41	368

L, Left; R, Right; BA, Brodmann area. Volume is measured in mm^3^. Coordinates reflect standard MNI space.

### Visual imagery

Whether early visual cortex, corresponding to Brodmann areas 17 and 18, participates critically in visual imagery has been a subject of much study (Kosslyn and Thompson, 2003). Visual input is rich in information, however, and we distinguish between different *types* of visual information. Importantly, a number of functionally specialized brain regions contain neurons that are preferentially tuned to different aspects of visual input: The lateral occipital complex (LOC) is specialized for shape processing (Sathian, 2005); neurons in area V4 are tuned to discriminate color (Bramão et al., 2010), and neurons in area V5/MT are critical in the perception of motion (Grèzes, 2001). Kosslyn and Thompson concluded that early visual cortex is involved in visual imagery, in the general sense, when the imagery task requires high-fidelity representations (Kosslyn and Thompson, 2003). An interesting extension to this question is whether the functional organization apparent in visual perception may be found in various subtypes of visual imagery. This is a strong test of the hypothesis that perceptual processes underlie imagery, as there is no reason that retrieval of stored visual representations—that is, visual information that has already been processed by the perceptual system—should *necessarily* require the involvement of these specialized brain regions. The following three analyses test whether form, color and motion imagery recruits the corresponding functionally specialized visual perception areas. Overlap between the form, color and motion ROIs was avoided by removing voxels appearing in the LOC ROI from the V4 and V5 ROI definitions.

### Visual form imagery

Minimum cluster size threshold in the ALE analysis of visual form imagery studies was set at 2384 mm^3^. Two hundred and forty eight foci from 21 experiments involving 218 participants contributed toward these results. For the purposes of this analysis, LOC was defined by the intersection of the Harvard-Oxford Cortical Structural Atlas definition of Lateral Occipital Cortex with the reverse inference map generated by Neurosynth (Yarkoni et al., 2011) for the term “LOC,” thresholded at *Z* > 5.39. The ALE analysis of all visual form imagery studies found seven clusters, bilaterally- but primarily left-distributed, reliably associated with visual form imagery (Table 4, Figure 2). There was overlap, centered at (*x* = −52, *y* = −62, *z* = −4: 631 mm^3^), between LOC and the smallest cluster centered at (*x* = −55, *y* = −59, *z* = −8). The 3dClustSim simulations determined that the volume of this overlapping region corresponded to a cluster size corrected threshold of *p* < 0.001 within the LOC ROI. Because the visual ROIs were adjacent to one another, the overlap of the visual form clusters with the color and motion ROIs was additionally assessed. The cluster overlapping with LOC additionally overlapped the visual color ROI definition by 126 mm^3^, which corresponded to a cluster size corrected threshold of *p* < 0.005 within the visual color ROI. No visual form ALE cluster overlapped with the visual color ROI.

### Visual color imagery

Minimum cluster size threshold in the ALE analysis of visual color imagery was set at 192 mm^3^. Eighty-one foci from seven experiments involving 76 participants contributed toward these results. For the purposes of this analysis, V4 was defined by the Juelich Histological Atlas definition of left and right V4. The ALE analysis of all visual color imagery studies found 4 left-lateralized clusters that were reliably associated with visual color imagery (Table 4, Figure 2). Overlap, centered at (left: *x* = −18, *y* = −82, *z* = −6: 42 mm^3^), was found between V4 and the largest cluster centered at (*x* = −13, *y* = −85, *z* = −4). The 3dClustSim simulations determined that the volume of this overlapping region corresponded to a cluster size corrected threshold of *p* < 0.05 within the V4 ROI. No visual color ALE cluster overlapped with either the visual form or visual motion ROIs.

### Visual motion imagery

Minimum cluster size threshold in the ALE analysis of visual motion imagery was set at 368 mm^3^. One hundred and ten foci from 10 experiments involving 97 participants contributed toward these results. For the purposes of this analysis, V5 was defined by the intersection of the Juelich Histological Atlas definition of left and right V5 with the reverse inference map generated by Neurosynth (Yarkoni et al., 2011) for the term “mt,” thresholded at *Z* > 5.39. The ALE analysis of all visual motion imagery studies found six clusters that were reliably associated with visual motion imagery (Table 4, Figure 2). Bilateral overlap between ALE clusters and V5 was noted (left: *x* = −42, *y* = −8, *z* = 14, 204 mm^3^; right: *x* = 47, *y* = −61, *z* = 1, 548 mm^3^). The 3dClustSim simulations determined that the volume of both overlapping regions corresponded to a cluster size corrected threshold of *p* < 0.001 within the V5 ROI. The cluster overlapping with right V5 additionally overlapped the visual form ROI definition by 262 mm^3^, which corresponded to a cluster size corrected threshold of *p* < 0.001 within the visual form ROI. The form and motion imagery clusters did not overlap within either of the ROIs, however there was a 244 mm^3^ overlap between form and motion imagery clusters centered in the right superior parietal lobule (*x* = 17, *y* = −66, *z* = 57; Brodmann area 7). The overlap between form and motion imagery activations in BA 7 is notable in light of the implication of this region in the integration of visual and motor information (Wolpert et al., 1998).

## General discussion

### Modality-general imagery

The first goal of this study was to identify the neural substrate underpinning modality-general imagery. Across all sensorimotor modalities, and many experimental paradigms, a core network emerged of brain regions associated with imagery. Activations were seen bilaterally in the general imagery analysis, and in some modalities (auditory, motor, gustatory, visual form and visual motion), but were primarily left-lateralized. It was noted earlier that perceptually-based representational theories assume that multisensory imagery underlies semantic retrieval. Others have suggested that the default-mode network, a well-defined network of brain regions more active during periods of rest than under cognitive load, may arise in part out of introspective processes, including imagery (Daselaar et al., 2010). Though the general imagery network bears a superficial resemblance to the resting state network described in the literature, it does not generally overlap with this network. The imagery network was derived from activations for contrasts of imagery greater than baseline. Activation of the resting state network would thus be precluded by definition. These results should not, therefore, be taken as evidence implying any particular property of the default-mode network. For example, a relative increase in imagery network activation may be apparent when resting state activity is compared to tasks that do not involve imagery.

### Modality-specific imagery

A second goal of this study was to identify the neural substrates underpinning modality-specific imagery, and assess the degree to which imagery in each modality recruited sensorimotor cortex. The ALE analysis of activation loci suggests that modality-specific imagery or knowledge retrieval for most modalities is associated with increased activation in corresponding sensorimotor regions. Though modalities differ with respect to the lateralization and extent of this recruitment, this suggests that modality-specific imagery generally recruits the corresponding primary perceptual areas. Whether these differences reflect differences in cognitive processing, or have behavioral implications remains unclear. For example, proportionally greater recruitment of perceptual regions may be associated with higher fidelity imagery, whereas greater recruitment of adjacent areas is associated with more abstract (e.g., linguistically-dependent) manipulations of imagery representations.

One challenge for this interpretation concerns the failure to show recruitment of primary sensorimotor perceptual cortices for the auditory and motor modalities. The ALE analyses showed imagery in these modalities does reliably recruit posterior superior temporal gyrus (STG) and premotor cortex, respectively. These results are consistent with Kosslyn et al. (2001) review finding that auditory imagery does not activate primary auditory cortex (A1), but does activate auditory associative areas. The same review concluded that motor imagery conditionally activates motor areas, but required a more liberal definition of motor area: Of the studies reviewed, most reported imagery-related activations in premotor cortex but not primary motor cortex. Posterior STG and premotor cortex have been associated with maintaining auditory and motor sequence representations, respectively (Ohbayashi et al., 2003; Arnott et al., 2005; Buchsbaum and D'Esposito, 2008). Thus, an alternative interpretation of imagery-related activations is that they reflect activations within memory systems for these modalities, and that these systems are situated adjacent to, rather than within primary auditory and motor cortices. This may indeed be the case, though such a conclusion rests on the sort of circular logic that highlights the centrality of the symbol grounding problem to understanding the neural bases of cognitive processes. It thus remains to be seen whether a satisfactory solution to the symbol grounding problem can be found for these imagery-related processes. These patterns are, however, suggestive of a modality-specific working memory system.

Though the question of whether visual imagery, in the general sense, recruits early visual cortex has been extensively studied (Kosslyn and Thompson, 2003), the more specific question of whether the functional distinction of color, motion and form perception is reliably found in visual imagery has remained unclear. A third goal of the present study was to determine whether similar functional specialization occurs in visual imagery. The present results indicate that, though visual imagery may activate early visual areas, imaginary color, motion, and shape processing is facilitated by upstream visual areas specialized for color, motion, and form perception, respectively. This parallel specialization during visual imagery is interesting in light of the fact that imagery involves the retrieval of stored representations. That is, imagery is based on information previously processed by the perceptual system. Nonetheless, imagery recruits brain regions involved in processing the original perceptual stream. To retrieve pre-processed rather than post-processed representations would thus be a sub-optimal strategy unless it conveys some other benefit. One possibility is that this processing reflect does not reflect processing of the raw visual stream. Rather, these regions may encode perceptual patterns that are reliably associated with information in other modalities. If this information is captured in the perceptual processing stream, it would be unnecessary to encode this information at higher levels of abstraction. Thus, imagery processes implied by perceptually-based representational theories may recruit these areas in order to generate more veridical multisensory representations.

Several crossmodal asymmetries were observed within the modality-specific imagery results. When the results of the motor and tactile imagery analyses are taken together, they suggest that motor imagery may imply a tactile component, but not the converse. This asymmetry may arise from the types of motor imagery tasks used: in more than half of the motor imagery tasks, the task implied imagery of an action on an object. This asymmetry would be predicted by the dependency of imagery on perceptual experience: one is often passively touched by objects (i.e., tactile perception without an associated motor response), but seldom acts on an object without also touching it. Similarly, an asymmetrical relationship existed among the three visual modality subtypes: First, form imagery clusters additionally overlapped the color ROI, but not *vice versa*. Second, motion imagery clusters additionally overlapped the form ROI, but not *vice versa*. This second asymmetry plausibly reflects our visual experience of moving objects: Form processing may be commonly implicated in motion processing because one typically perceives motion of an object with form. The converse relationship does not seem quite as strong, as we regularly encounter inanimate forms that do not move. In contrast, the apparently consistent recruitment of primary color processing regions during form imagery, but not the converse, is puzzling. We do not usually experience fields of color, but instead see colored objects, or *forms*. On the other hand, we do regularly experience well-defined forms without any associated color: square vs. oval windows, for example. The observed asymmetry would thus appear to be the reverse of what one would expect on the basis of real-world experience. One possibility is that it reflects an interaction between a statistical artifact of the number of form imagery studies and the proximity of the two regions of interest. More visual form imagery studies were conducted with more participants, generating more extensive ALE maps, with a higher probability of overlapping an adjacent ROI. Alternatively, it may reflect a real property of the systems involved in color and form imagery, though that remains a subject for future investigation.

### Modality-specific imagery and perceptually-grounded representations

Finally, and perhaps most importantly for investigations of perceptually-grounded representations, in no modality were imagery clusters restricted to brain regions immediately involved in perception. Those clusters that did overlap with primary somatosensory regions generally extended beyond these areas. In contrast to perception or imagery-based accounts of knowledge representations, amodal models of semantic memory assume concept knowledge is maintained as an abstraction bearing no connection to perceptual processing (Pylyshyn, 1973; Tyler and Moss, 2001). It is no less reasonable to suppose that a modality-specific representational system encodes information in sensory association areas, but not necessarily in primary sensorimotor areas. This perspective is consistent with Thompson-Schill's review of neuroimaging studies of semantic memory (Thompson-Schill, 2003), which concluded that the literature supported a distributed modality-specific semantic system, but that “studies which have directly compared semantic retrieval and perception have consistently found an anterior shift in activation during semantic processing” (p. 283).

The present meta-analysis suggests that a perceptually-grounded representational system recruits primary sensory cortex to a modest and varying degree, but that processing relies greatly on upstream (though not necessarily anatomically anterior) unimodal convergence zones (Binder and Desai, 2011; McNorgan et al., 2011). These regions tend to be adjacent to their associated perceptual areas, and integrate downstream perceptual codes into somewhat more abstract (but perceptually-grounded) representations. This account would be consistent with the distribution of modality-specific imagery activations about primary somatosensory cortices, and with the theoretical ties between modality-specific representations and imagery. This interpretation would also be consistent with a recent investigation of visual imagery and memory by Slotnick et al. (2011) in which the authors concluded that “visual memory and visual mental imagery are mediated by largely overlapping neural substrates in both frontal-parietal control regions and occipital-temporal sensory regions” (p. 20). These results suggest that neuroimaging investigations of perceptually-based knowledge might pay particular attention to primary sensorimotor areas also implied in imagery, but also should consider contributions of other brain regions supporting imagery processes.

## Conclusions

Though neuroscientific studies of imagery have proliferated over the last decade, not all forms of imagery have been investigated to the same extent—imagery of the chemical senses and tactile imagery appear to be relatively underrepresented. Some of the questions posed here may not be adequately answerable without further study in these imagery domains. Similarly, the present review omits studies of imagery in other domains, such as emotional, temporal or spatial imagery, which may be more abstract forms of meta-imagery involving the integration of multiple modalities or function as representational primitives.

Finally, these results are generally consistent with the assumption that mental imagery underlies representational knowledge, though the matter is far from resolved. These considerations point toward a need for further investigation in the imagery domain. These efforts will help relate cognitive processes to one another and to arrive at a fully grounded model of cognitive processing.

### Conflict of interest statement

The author declares that the research was conducted in the absence of any commercial or financial relationships that could be construed as a potential conflict of interest.
